# Mechanisms Underlying Graft Union Formation and Rootstock Scion Interaction in Horticultural Plants

**DOI:** 10.3389/fpls.2020.590847

**Published:** 2020-12-10

**Authors:** Aatifa Rasool, Sheikh Mansoor, K. M. Bhat, G. I. Hassan, Tawseef Rehman Baba, Mohammed Nasser Alyemeni, Abdulaziz Abdullah Alsahli, Hamed A. El-Serehy, Bilal Ahmad Paray, Parvaiz Ahmad

**Affiliations:** ^1^Department of Fruit Science, Sher-e-Kashmir University of Agricultural Sciences and Technology of Kashmir, Srinagar, India; ^2^Division of Biochemistry, Faculty of Basic Science, Sher-e-Kashmir University of Agricultural Sciences and Technology of Kashmir, Srinagar, India; ^3^Botany and Microbiology Department, College of Science, King Saud University, Riyad, Saudi Arabia; ^4^Department of Zoology, College of Sciences, King Saud University, Riyad, Saudi Arabia

**Keywords:** grafting, incompatibility, phytohormones, callus bridge, rootstock-scion

## Abstract

Grafting is a common practice for vegetative propagation and trait improvement in horticultural plants. A general prerequisite for successful grafting and long term survival of grafted plants is taxonomic proximity between the root stock and scion. For the success of a grafting operation, rootstock and scion should essentially be closely related. Interaction between the rootstock and scion involves complex physiological-biochemical and molecular mechanisms. Successful graft union formation involves a series of steps viz., lining up of vascular cambium, generation of a wound healing response, callus bridge formation, followed by vascular cambium formation and subsequent formation of the secondary xylem and phloem. For grafted trees compatibility between the rootstock/scion is the most essential factor for their better performance and longevity. Graft incompatibility occurs on account of a number of factors including of unfavorable physiological responses across the graft union, transmission of virus or phytoplasma and anatomical deformities of vascular tissue at the graft junction. In order to avoid the incompatibility problems, it is important to predict the same at an early stage. Phytohormones, especially auxins regulate key events in graft union formation between the rootstock and scion, while others function to facilitate the signaling pathways. Transport of macro as well as micro molecules across long distances results in phenotypic variation shown by grafted plants, therefore grafting can be used to determine the pattern and rate of recurrence of this transport. A better understanding of rootstock scion interactions, endogenous growth substances, soil or climatic factors needs to be studied, which would facilitate efficient selection and use of rootstocks in the future. Protein, hormones, mRNA and small RNA transport across the junction is currently emerging as an important mechanism which controls the stock/scion communication and simultaneously may play a crucial role in understanding the physiology of grafting more precisely. This review provides an understanding of the physiological, biochemical and molecular basis underlying grafting with special reference to horticultural plants.

## Introduction

**FIGURE 1:**
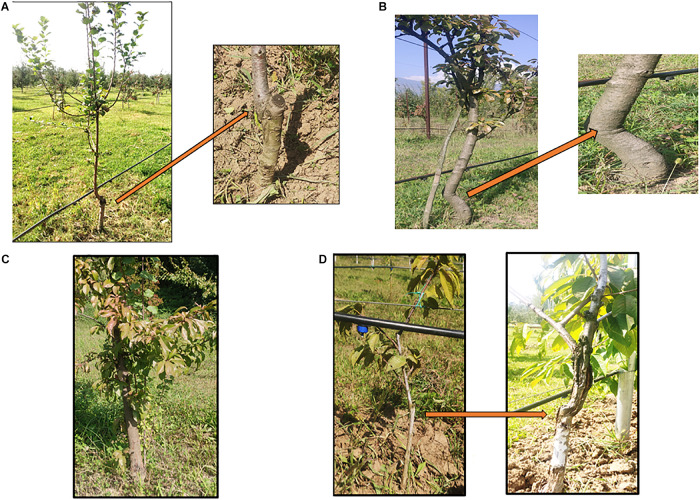
**(A)** Grafted Apple (Rootstock- M9, grafting method-Wedge grafting, Age-7 years); **(B)** Grafted Pear (Rootstock- Quince- C, grafting method- Wedge grafting, Age-8 years); **(C)** Plum (Rootstock- Seedling, grafting method- Tongue grafting, Age-5 years); **(D)** Cherry (Rootstock- Gisela 5, Grafting method- Tongue grafting, Age-6 years).

Grafting has been performed in agriculture since the beginning of civilization. Historical records have revealed that ancient Chinese and Greeks have been practicing it since 1560 B.C. (Melnyk and Meyerowitz, 2015). Since fruit and nut trees are difficult to propagate by cuttings, grafting is used for their propagation. Moreover, the superiority and quality of the grafted crops further led to widespread adoption of this technique. It is a well-established practice which makes it possible to physically join two or more genetic entities in a single tree to influence the productivity characters of a tree favorably and facilitates asexual propagation in horticultural crops like apple, pear, plum and cherry (Figures 1A–D; Kumari et al., 2015). A “*de novo*” formed meristematic area must develop between scion and rootstock for a successful graft union. The scion becomes the new shoot system and the rootstock (under stock, stock) forms the root system of the grafted plant. Scions are selected based on yield related traits and are generally grafted over specific rootstocks having the ability to survive the biotic and abiotic components of the environment. Rootstock mostly influences scion vigor and its water relations. Grafting is usually practiced in perennial horticultural trees with the main aim to reduce vegetative growth and shorten the juvenile period. Additional benefits of grafting include dwarf tree structures to increase the planting density per unit area and hence productivity simultaneously with minimal investments in orchard cultural practices like pruning, pest and foliar disease control. For an efficient root system to develop the rootstock and scion compatibility plays a crucial role (Goldschmidt, 2014; Warschefsky et al., 2016). However, rootstock and scion compatibility vary to an extent that even the closely related species might not be compatible therefore, it becomes necessary to evaluate the compatibility before grafting a particular scion into a rootstock (Lee et al., 2010; Guan et al., 2012). Success of a grafting operation depends on the strength of the union formed. Stronger unions result in successful grafting operation. On the contrary, weaker unions lead to graft failure and in adverse cases the trees may fall apart. Graft union formation depends on a number of factors viz., molecular pathways and physiological/biochemical responses. Lot of effort has been put into studying the physiological mechanism of union formation, causes and consequences of graft incompatibility and also as to how molecules are being transferred across the graft unions to reveal the mechanisms responsible for inducing the phenotypic changes by grafting. In this review we not only get an idea about the fundamental mechanism of graft union formation, graft incompatibility: its types, mechanism and causes, but it also makes some of the critical molecular and physiological mechanisms associated with grafting much easier for us to understand.

## Grafting Techniques in Fruit Trees

**FIGURE 2:**
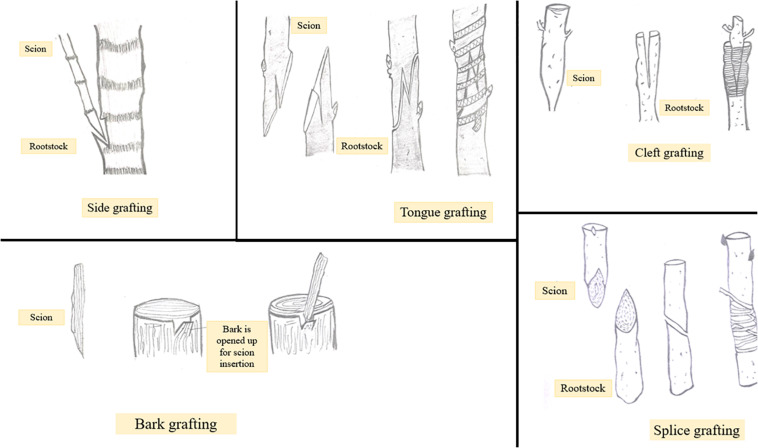
Different types of grafting like side, tongue, cleft, bark, and splice.

Grafting has become immensely important in view of improving cultivation especially of fruit and vegetable crops. Besides this, grafting tends to improve adaptability and resistance of plants to different environments and stresses (Kumar et al., 2017). The latter can be achieved by using a suitable rootstock. However, not every grafting operation is successful. Apart from a number of factors including stock scion combination used, season, temperature, etc., the technique of grafting followed is the most important one to determine the degree of grafting success (Soleimani et al., 2010). Grafting techniques include side, tongue, cleft, bark, and splice grafting methods (Figure 2). Among these cleft grafting also known as wedge grafting is the most commonly used method. Additionally, budding is also a form of grafting in which the scion size is reduced to a small piece of stem with one or more axillary bud attached to it. In general, success of a grafting operation depends on combining anatomical structures of the stock and scion, so much so that if there is some dislocation of vascular elements, weak or distorted unions may get formed eventually leading to graft failure (Wang, 2011). Thus, the choice of an appropriate grafting method is critical to ensure proper contact between stock and scion and to avoid the formation of incompatible or weak graft unions. Depending upon the existing environmental conditions the success of any grafting method may vary from one crop to another. Maximum success percentage i.e., 100% was obtained in mango by following cleft grafting technique in the month of June or March. On the other hand, Allan et al. (2010) reported that in papaya side grafting brings about 80% success rate while Nguyen and Yen (2018) recommended cleft grafting using 1-month old rootstocks as the best method for maximum grafting success in papaya. Maximum graft success in plum i.e., 9.67 out of 10 grafts was achieved by following cleft grafting in April suggesting it to be the commercial method for grafting (Mozumder et al., 2017). In walnut wedge grafting was found comparatively superior to tongue grafting in terms of sprouting percentage, graft union success, and plant growth (Srivastava, 2012). The influence of grafting technique on plant growth has been studied in peach cultivar Shan-e-Punjab grafted on wild peach rootstock. Different grafting methods including tongue grafting, chip budding and T budding were followed. Tongue grafted plants showed maximum sprouting percentage, graft success, plant height, girth and number of branches. The results indicate that tongue grafting is the best method of propagation for peach variety Shan-e-Punjab (Sharma et al., 2018). Bud take and bud sprouting were found to be earliest when T budding (with wood) was performed in cherry. Also, maximum shoot length and the highest number of leaves and lateral branches were obtained with T budding (with wood) compared to the other two methods i.e., T without wood budding and chip budding (Yazdani et al., 2016). Graft take success in apple cultivars grafted on different rootstocks was evaluated and it was found that the cultivar Gala mast grafted on crab apple rootstock by means of bench grafting showed maximum graft take success as well as prominent growth (Fazal et al., 2014). Whip and cleft grafting methods have been reported to be the most promising ones for the asexual propagation of Jabuticabeira Acu grafted on rootstocks belonging to other species of the same family (Cassol et al., 2017). Depending upon the research purposes other grafting methods like *in vitro* grafting have been introduced. Besides being time consuming, the planting material used in the conventional system of plant propagation is not healthy. To overcome these problems application of *in vitro* techniques is an effective alternative. One such application is shoot tip grafting or micrografting. Micrografting involves *in vitro* placing of shoot tip as an explant on a decapitated rootstock grown from a seed (Hussain et al., 2014). Micrografting protocols have been developed for many fruit crops including grapes (Aazami and Bagher, 2010), walnut (Wang et al., 2010), almond (Yıldırım et al., 2013), etc. Rafail and Mosleh (2010) reported that in *in vitro* grafting of apple and pear, homografting was relatively more successful compared to heterografting and an increase in micrograft success was noticed from 30 to 90% in pear (cv. Aly-sur on Calleryana pear) and 40 to 90% in apple (cv. Anna on MM106) with increasing benzylaminopurine (BAP) concentration from 0 to 2.0 mg/L. Patharnakh shoot tips were propagated *in vitro* on Kainth rootstock. Graft success and vigor was found to be maximum by following wedge grafting method and using 5–10 mm scions and M2 (MS liquid media +20 g/l sucrose) media (Rehman and Gill, 2014). Hetero-grafting allows the alteration of important plant processes including water uptake, nutrient absorption, hormonal signaling and enzyme activity. Cookson and Ollat (2013) reported that it is heterografting and not homografting which affects the gene expression pattern in shoot apical regions. Stress response genes were upregulated at the graft interface of heterografts compared to homograft’s indicating that the cells at the graft interface have the ability to recognize and function differently as soon as they come in contact with a self or non-self-grafting partner (Cookson et al., 2014). Comparative analysis of differentially expressed genes in homo and heterografted tomato seedlings was carried out and it was found that in heterografts healing process was slightly slow compared to homograft’s and several genes involved in oxidative stress were significantly up-regulated in the scion of heterografted tomato (Wang et al., 2019). Gene expression studies in homo and heterografts of grapevine revealed upregulation of genes involved in the synthesis of cell wall, wound responses, hormone signaling and other metabolic pathways in homograft’s, while in heterografts stress responsive genes were up-regulated at the graft interface (Clemente Moreno et al., 2014). Studies on efficiency of grafting techniques and time of grafting have been conducted and standardized for different areas (Ghosh and Bera, 2015). This information may help in determining ideal grafting time for quick multiplication of fruit crops to enhance quality fruit production. In view of the same, application of *in vitro* grafting techniques for propagation of fruit crops can be considered a relatively sustainable alternative to conventional methods of propagation. Micrografting technique has a great scope in plant improvement and their large-scale propagation. Production of virus free plants is one important advantage of this technique due to which it finds application in fruit crop propagation. In addition to this, prediction of graft incompatibility has been possible through micrografting. Grafting operation can be conducted at any time of the year through micrografting. Due to an adequate number of advantages this technology has great potential to be practically used by researchers and nursery growers.

## Graft Union Formation

**FIGURE 3:**
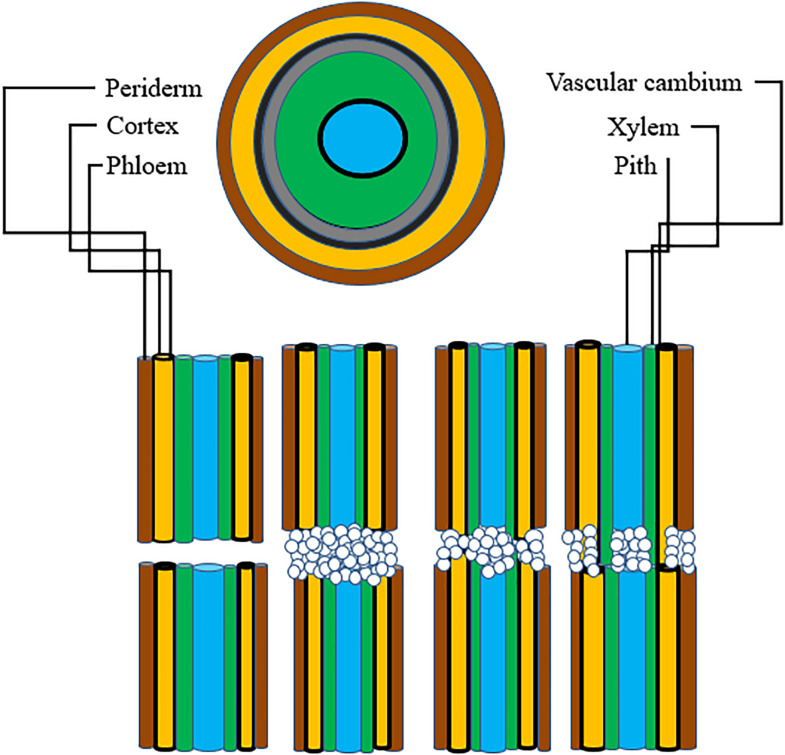
Represents stages of graft union formation: Stage 1, Parenchymatous tissue divides to form callus cells; Stage 2, Xylem vessel formation; Stage 3, Formation of vascular cambium across the graft union linking the two partners; Stage 4, Secondary xylem and phloem dedifferentiate across the graft union establishing sufficient vascular continuity for plant growth [Hartmann and Kester, (2002)].

For grafting to be successful, a number of complex biochemical and structural processes are involved. The latter result in establishing a connection between the root-stock and scion. Adhesion of parenchyma is the first step for union formation followed by formation of vascular elements and their differentiation into xylem and phloem. Formation of vascular connection between the stock and scion during wound healing is of utmost importance as the wound given to the stock and scion during grafting causes disruption of the vascular system in plants (Asahina and Satoh, 2015), hence connecting up of the vascular system is required to facilitate water uptake as well as to ensure nutrient transport to the graft junction. In addition to this, vascular reconstruction enables macromolecules to be transported across the graft union (Harada, 2010). This specifies that vascular differentiation is imperative for grafting success during the process of wound healing. Five histological stages are reported to come about during graft union formation in rootstock scion combinations: (1) formation and orientation of necrotic layers, (2) callus cell proliferation, (3) formation of callus bridge at the graft interface, (4) vascular cambium formation and (5) vascular tissue reconstruction between the stock and scion (Figure 3). Except for the outer cortex necrotic layers tend to disappear by the cellular activities in the callus. In most of the cases the portion of necrotic layers in the outer cortex gets transformed into bark (Yildirim et al., 2010). The process of graft union formation is temporally separated. Herbaceous plants take relatively shorter time for the successful formation of a graft union compared to trees. Time required for graft union formation in the *Arabidopsis* micrograft system is 7–12 days (Turnbull, 2010). On the contrary, trees take several months to form a union at the graft interface (Olmstead et al., 2006). The process of vascular reconnection during the formation of a graft union has been studied in *Arabidopsis thaliana*. Attachment of tissues on either side of the cut surfaces, establishment of a connection between the phloem cells of rootstock and scion, resumption of root growth, and connection between xylem vessels were found to be temporally separated. It was found that at first the two parts get attached to each other, this is followed by connecting up of phloem about 3 days after germination (DAG), growth of the roots gets resumed at this stage around 5 DAG, and at 7 DAG xylem vessels get re-joined. By analyzing the pattern of cell division and tissue regeneration at the graft junction it was observed that cells at first showed an asymmetrical pattern of cell division and differentiation but as soon as a contact establishes between the stock and scion, they tend to lose this asymmetry and the vascular connection gets re-established (Melnyk et al., 2015). Melnyk et al. (2018) in their subsequent study explained in detail the differential expression and upregulation of many genes during the union formation leading to vascular regeneration. They described that genes are expressed asymmetrically at the graft junction in *Arabidopsis* hypocotyl grafts. This differential expression of genes was observed on account of abundant carbon concentration in the scion and less carbon in the rootstock, till the phloem was reconnected. At the graft union, genes associated with the formation of vascular tissues were upregulated, thereby activating a recognition mechanism between the stock and the scion. Different stages of union formation at the graft interface in tomato seedlings revealed that a number of structures appeared to interconnect the stock and scion 8 DAG, vascular bridges appeared at 11 DAG and connection between the root-stock and scion got completely established 14 DAG (Fan et al., 2015). On the other hand, histological stages of development of graft union in spur type apple varieties grafted on different apple rootstocks revealed that ample amounts of callus boomed in all the stock-scion combinations. Formation of cambium and reconnection of vascular cells was apparently successful in 90-day old grafts. Callus bridge filled the stock and scion gap on 120th day and it continued for a few more days, following which xylem and phloem strands bridged the union (Polat et al., 2010). Also, study of changes at the graft union in cashew by Mahunu et al. (2012) revealed that 30 DAG the necrotic layer disappeared, coinciding with the enlargement of callus, while adhesion of stock and scion occurred at 60 DAG. At 98 DAG cambial connections and healed unions were visible. However, in case of unsuccessful graft combinations at 98 days after grafting, a gap between the cells of stock and scion was noticed indicating that union formation is the key factor for graft success and further growth of the grafted plant. Since grafting puts a considerable amount of stress on plants, it is associated with the stimulation of a number of wounding responses such as production of ROS (reactive oxygen species), upregulation of certain genes providing stress resistance, synthesis of enzymes and other chemical substances. These compounds eventually trigger the formation of wound induced callus. Furthermore, the growing callus is sustained by production and stimulation of specific metabolites. Study of transcriptional changes at the graft interface in grapevine at 3 and 28 DAG revealed differential expression of certain genes involved in the synthesis of cell wall and formation of vascular elements. Expression of these genes was particularly upregulated at the graft interface compared to the rootstock which resulted in the identification of genes specific to the graft interface (Cookson et al., 2013). To identify the compounds involved in callus formation, Prodhomme et al. (2019) conducted metabolite profiling in grapevine. The study revealed increased production of amino acids (basic and branched chain) as well as accumulation of stilbene compounds at the graft interface. Additionally, the union formation was associated with increased activity of two enzymes *viz*., *PAL* and *NI* at the graft interface compared to the surrounding tissue. All these metabolic modifications together support callus growth and serve as a source for the identification of potential markers for selection in breeding programs. Despite these findings, we still lack the understanding of how the two components i.e., stock and scion actually establish a physical connection, integration of the vascular tissues, role of plasmodesmata in union formation, and material exchange at the graft interface. Thus, advanced research is needed to address these basic questions. This can be done by using fluorescent markers and correlative light-electron microscopy techniques (Gautier et al., 2019).

## Genetic Limits of Grafting

**FIGURE 4:**
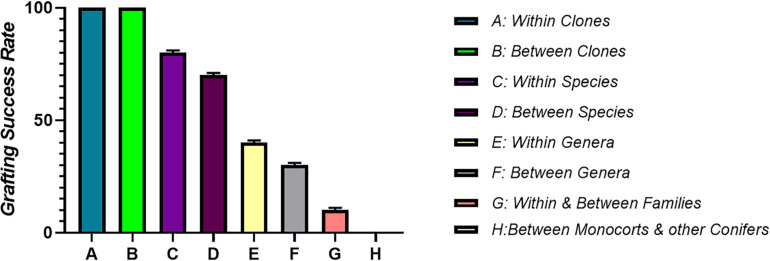
It shows correlation between taxonomic proximity and graft compatibility. Grafting between closely related plants is comparatively more successful than distantly related ones. Maximum grafting success is achieved by performing grafting within or between the clones. Grafting success goes on decreasing as the plants become less related taxonomically.

A prerequisite for graft compatibility is taxonomic proximity. Autografts are taxonomically quite close and thus are expected to be always compatible. When grafting is performed within the same species it forms compatible combinations, if the grafting partners are from different species but the genus to which they belong is same, grafts are more or less compatible, intra familial grafts are rarely compatible, while inter familial grafts are essentially unsuccessful due to incompatibility (Mudge et al., 2009). Therefore, the taxonomic proximity between the grafting partners is essential for the successful re-establishment of both the rootstock and scion fused together. It is a very well-known fact that presence of vascular cambium is a prerequisite for grafting. Vascular cambium due to its meristematic activity divides to form xylem and phloem during the secondary growth resulting in increased plant diameter (Spicer and Groover, 2010). Monocots cannot be grafted, moreover grafting of a monocot plant onto a dicotyledonous plant is also not possible. This is because vascular bundles in monocots are scattered and they lack cambium, which is a basic requirement for graft union formation. The parallel venation in the leaves of monocots indicates that the veins do not interconnect to each other like they do in dicots. Thus, it is the lack of vein connection in stem and leaves of monocot plants which makes grafting in monocots an impossible task (Figure 4). Plants that are closely related have a good chance of successful union formation compared to the remotely related ones. Such plants have less or no chance of successful graft union formation. In order to achieve maximum success, grafting should be performed between or within the clones (Kumar, 2011). However, successful interfamilial graft combinations between *Nicotiana benthamiana* (Nb) and *Arabidopsis thaliana* (At) have been reported where the growth of Nb scion was slow but distinct at the same time (Notaguchi et al., 2015). During the course of time, plants have developed a specialized haustorium that pierces into the host plant to derive nutrients by means of tissue adhesion and this property of cell to cell adhesion can be used to develop interfamilial grafts (Westwood et al., 2010). Natural tendency for cell to cell adhesion with plants belonging to different families including vegetables, fruits trees as well as monocots is found in *Nicotiana*. Here, the reconstruction of vascular structures follows the normal pattern as in case of intrafamilial grafting. A transcriptomic study revealed the upregulation of β-1,4-glucanases followed by graft adhesion in inter as well as intra familial grafts. The use of *Nicotiana* stem, an interscion produced tomato fruits on rootstocks belonging to different families. Therefore, the mechanism of cell to cell adhesion can be used to modify plant grafting techniques and to develop graft combinations which are otherwise difficult to obtain (Notaguchi et al., 2020).

## Graft Incompatibility: Types and Determining Techniques

Graft incompatibility is generally referred to as inability of the stock and scion to bind together to form a successful graft union. Lack of compatibility between the rootstock and scion is the major limiting factor in propagation of fruit trees, particularly stone fruits (Zarrouk et al., 2006). Graft incompatibility is therefore a critical issue for breeding rootstocks of fruit trees and longevity of an orchard (Hossein et al., 2008). It leads to the formation of unhealthy trees, breakage at the graft union and premature death of grafted plants (Zarrouk et al., 2006). The arrival of these symptoms could take a number of years (Guclu and Koyuncu, 2012). Thus, to ensure a successful graft union the selection of a mutually compatible scion/rootstock combination is important (Goldschmidt, 2014).

**FIGURE 5:**
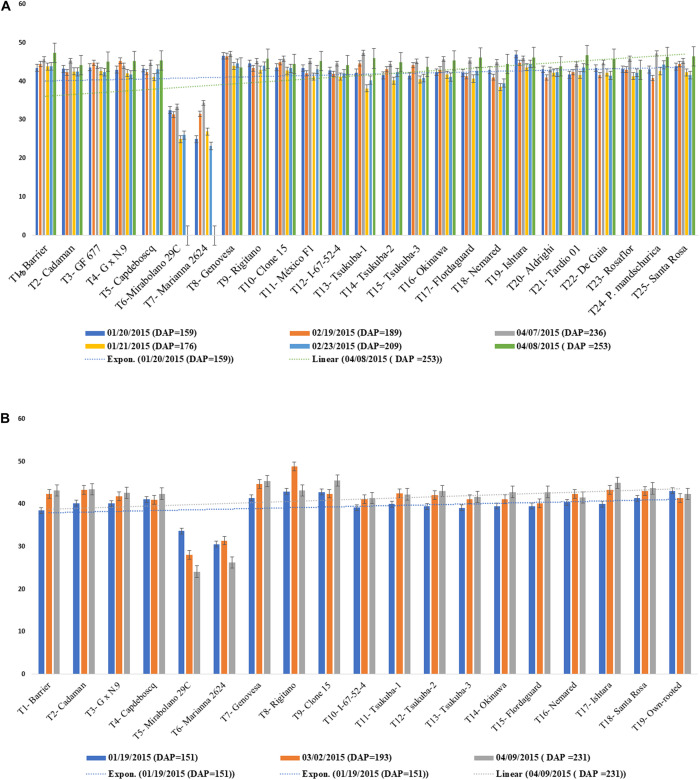
**(A)** SPAD values for Jade and Maciel (scion cultivars) grafted onto a range of clonal rootstocks. **(B)** SPAD values BRS-Kampai scions grafted on a range of clonal rootstocks (Neves et al., 2017).

Graft incompatibility is broadly categorized as: translocated and localized (Zarrouk et al., 2006). In case of “translocated” incompatibility, symptoms are observed at early stages of plant development. Scion and root growth tend to terminate at a very early stage, reduced carbohydrate translocation at the union, shriveling of leaves, leaf chlorosis leading to leaf reddening and early leaf drop are commonly observed symptoms. Translocated incompatibility can be evaluated using the soil plant analysis development (SPAD) chlorophyll meter which measures the chlorophyll content and nitrogen status of plants. Low SPAD index values indicate restricted carbohydrate assimilation and nitrogen uptake due to translocated incompatibility (Zarrouk et al., 2006). Significantly lower SPAD index values were observed for rootstocks “Mirabolano 29C” and “Marianna 2624” in the three scion cultivars, ultimately resulting in death of the trees owing to incompatibility between the graft partners. The rootstocks showing translocated graft incompatibility symptoms like reddening of leaves, excessive leaf curling, leaf drop, etc., with scion cultivars died 5 months after planting. SPAD index values did not decrease in other scion/rootstock combinations after 9 months of planting, indicative of their compatibility (Figures 5A,B; Neves et al., 2017). Thus, SPAD chlorophyll meter serves as an effective and non-destructive tool for the prediction of incompatible graft combinations.

**FIGURE 6:**
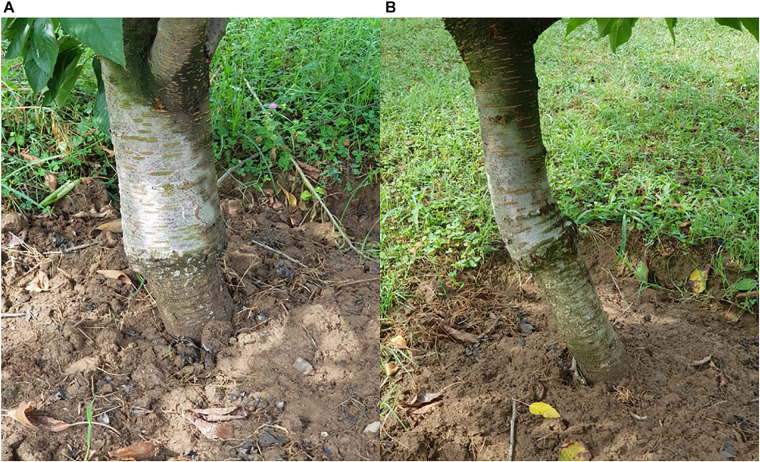
Overgrowth above the union caused by blockage of photosynthates, translocating from the scion into the stock, when sweet cherry is grafted onto sour cherry [**(A)** Age 10–12 years; **(B)** Age 7–8 years].

Localized incompatibility, on the other hand, leads to malformation at the graft union due to physiological and morphological changes taking place which eventually results in impaired union formation and in severe cases the tree might fall apart at the junction after some years of grafting (Errea, 1998). The alterations associated with localized incompatibility include disruption of vascular cambium, lower rate of tissue differentiation, lignification may not take place efficiently and disruption of vascular continuity. These changes might cause the graft union to rupture (Zarrouk et al., 2006, 2010; Pina et al., 2012, 2017). The most known example of localized incompatibility is between pear and quince, when pear cultivars are used as scion and quince as rootstock, prunasin, a cyanogenic glycoside generally present in quince but absent in pear is translocated into the phloem cells in pear scion, where the pear enzymes disrupt the prunasin in the region of graft union, producing hydrocyanic acid as one of the products of decomposition. Hydrocyanic acid obstructs the actively dividing cambial cells at the graft union and also disrupts phloem tissues at and above the graft union. Restriction in water flow and mineral/metabolite translocation across the union consequently kills quince phloem as well (Gur et al., 1968). Early and correct forecast of graft incompatibility is of utmost significance because the unwanted incompatible combinations could be avoided while the desirable compatible ones could be selected (Petkou et al., 2004; Gökbayrak et al., 2007). Standardized methods for evaluation of compatibility between the rootstock and scion would be of great use to the breeders while using a particular rootstock and scion for grafting (Pina et al., 2017). In several apricot combinations grafted on prunus rootstocks, graft incompatibility resulted in breakdown of the trees at the union years after planting, therefore an early selection process could help in detecting a comparatively compatible combination. Analysis of the phenol content at the graft union can be used as a technique for the estimation of graft incompatibility (Dogra et al., 2018). Callus formation is of utmost importance for stock and scion to be compatible and grafting to be successful. In grapevine it was found that it is not the graft take rates but the status of callus formation at 21 DAG which is an indicative of compatibility between the stock and scion. Moreover, analysis of leaf chlorophyll content can also serve as an efficient means to estimate the compatibility (Tedesco et al., 2020). The difference in the quality and quantity of phenol in the stock and scion can point toward metabolic dysfunctions at the graft union (Errea, 1998) and can serve as a biochemical marker to predict graft incompatibility (Musacchi et al., 2000). Histological studies of callus formation and cell alignment have made it possible to predict the compatibility of any combination way before the symptoms appear (Errea and Borruey, 2004). Additionally, the findings of Guclu and Koyuncu (2012) revealed that by using peroxidase activity as a technique it is possible to predict graft incompatibility before the grafting operation is conducted particularly in those combinations that might show delayed incompatibility symptoms (Figure 6). Furthermore, electrophoresis method and X-ray tomography can serve as important tools for assessment of graft quality and success (Dogra et al., 2018). The complex mechanisms involved in the incompatibility reaction between the stock and scion have been studied in many ways, however, still many processes remain unclear therefore, advanced research is needed to completely understand the physiology of graft incompatibility especially in perennial plants.

## Role of Phenolic Compounds in Graft Incompatibility

Incompatibility is allied with complex biochemical and physiological interactions between the grafting partners (Pereira et al., 2014). Pina and Errea (2008), suggested that graft compatibility/incompatibility response could be related to the protein UDP-glucose pyrophosphorylase. Phenolic substances play a crucial part in plants and are one of the most important compounds that determine the rootstock-scion interactions. Errea (1998) believed that quality and quantity of phenols in rootstock-scion parts indicated why the rate of metabolic activities is low at the graft union. Mng’omba et al. (2008) presented that the combinations which are apparently less compatible possessed high concentrations of phenolic compounds than the compatible ones. r-coumaric acid was present in a huge amount in relatively less compatible combinations. Therefore, phenols particularly r-coumaric acids and flavonoids resulted in weak union formation at the graft junction. This is the peculiar sign of graft incompatibility. Pear stock-scion combinations were found associated with profuse amounts of arbutin in phloem above and below the graft union, after that procyanidin B1 and chlorogenic acid. Compatible combinations had greater arbutin levels above the graft junction, whereas in the incompatible combinations of “Williams” on quince MA high arbutin concentration was recorded at the lower side of the graft union. In all the cultivars under study, arbutin content was found to be highest below the graft union specifying that it’s not just catechin and procyanidin B1, but also arbutin and a number of flavonols may possibly serve as a cause for graft incompatibility (Hudina et al., 2014). Several studies have shown that phenolic compounds in incompatible combinations move from vacuole to cytoplasm and cause inhibition of lignification which is required during early stages of establishment of scion–stock connections. The cell wall of xylem vessels are dynamic in nature composed of phenolic compounds (for example, lignins), minerals, polysaccharides and proteins (Liu, 2012; Herrero et al., 2014). These phenolic compounds can serve as important markers for determining compatibility between different graft combinations (Prabpree et al., 2018). The role of different cyanogenic glycosides (CGs) in the incompatibility reaction of Prunus has been evaluated in different graft combinations belonging to prunus species *viz., Prunus persica* and *Prunus mume* and ungrafted genotypes. The incompatible graft combinations were found to have greater prunasin levels and activity of the enzyme phenylalanine ammonia lyase (*PAL*) was also higher in rootstock. Additionally, the scion and stock were found to have a moderately higher concentration of total phenolic compounds with high antioxidant activity. Differences in concentration of CGs, primarily prunasin, was found to be responsible for the incompatibility between *Prunus persica* and *Prunus mume* (Pereira et al., 2018). Therefore, grafting between such plants with great differences in CG concentrations may end up in generating incompatibility reactions between the partners (Pereira et al., 2015). Plant hormones, especially auxins determine the compatibility of a rootstock-scion combination by interacting with phenolic compounds. Incompatibility has been associated with increased levels of phenolic compounds above the graft union which adversely affect the auxin transport (Errea, 1998). Low auxin concentration in incompatible combinations in turn affect the differentiation of vascular tissues and lignification (Aloni, 2010; Koepke and Dhingra, 2013). All these changes will lead to the formation of weak unions which may cause huge economic losses to the growers. More information about the compounds responsible for inducing graft incompatibility is needed. This knowledge is necessary for the development of molecular markers for rootstock breeding (Gainza et al., 2015).

## Hormonal Control of Graft Union

**FIGURE 7:**
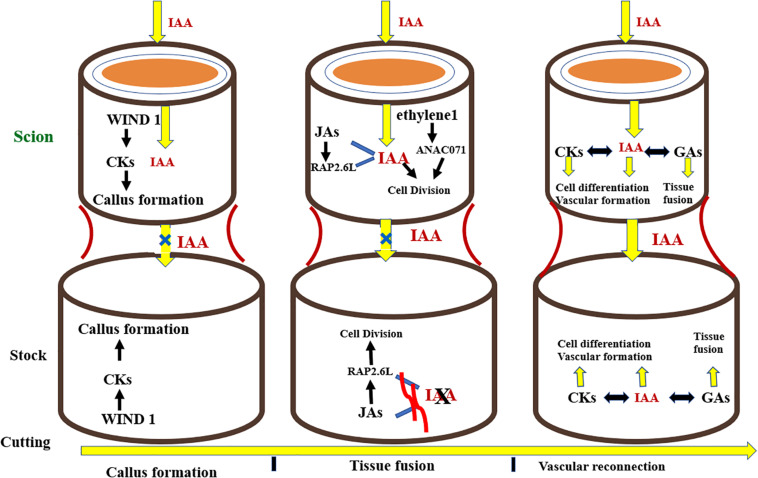
Hormonal signaling taking place at the graft interface during the grafting process. CKs, cytokinins; IAA, auxin; 534 JAs, Jasmonic acids; GAs, Gibberellins.

The development of successful grafts involves some fundamental steps in the following pattern: phloem tissue reunion, root growth, and xylem tissue reconnection (Melnyk et al., 2015). The key event involved in the formation of a graft is the joining of vascular tissues between the rootstock and scion. Grafting first and foremost causes the discharge of pectins from cells at the graft union which makes the rootstock and scion adhere together. Dedifferentiated cells at the union form callus at the graft interface, these cells then interdigitate and form a connection via plasmodesmata. The vascular tissues differentiate together with the callus at the grafting site giving rise to phloem which is succeeded by reconnection of xylem tissue (Jeffree and Yeoman, 1983; Melnyk et al., 2015; Ribeiro et al., 2015). Plant hormones commonly known as phytohormones regulate all phases of growth and development in plants, from embryogenesis to reproductive development. They standardize the response of plants to a wide range of biotic and abiotic stresses. Additionally, phytohormones regulate the physiological processes taking place at the site of graft union. Phytohormones facilitate plants to combat the stress induced by grafting. Aloni (1995) found that relatively less concentration of indole-3-acetic acid (*IAA*) encouraged phloem differentiation, but higher levels brought about the differentiation of xylem. Similarly, in grafting trials, auxins are an essential group of elements resulting in the formation of compatible graft unions (Figure 7). Auxins are produced from the vascular strands of the stock and scion, which bring about the vascular tissue differentiation, hence working as a growth regulating substance (Aloni, 1987; Mattsson et al., 2003). Plant hormones are translocated from source to sink as signal molecules influencing growth of cells and differentiation of vascular tissues, especially at the graft crossing point (Aloni, 2010). Kümpers and Bishopp (2015) demonstrated that phytohormones regulate the complex physiological interaction between scions and rootstocks in *A. thaliana*. Along these lines, they may be well thought-out candidates in the scion–rootstock communication both above and below the grafted interface (Kondo et al., 2014). Nanda and Melnyk (2018) inspected the function of eight plant hormones for the period of grafting to determine their role in healing of cut surfaces and vascular tissue differentiation in plants at the graft interface (Melnyk, 2017). They concluded that every known plant hormone controls the vascular tissue differentiation during the period of graft union formation. Nonetheless, auxin is the principal regulator of vascular differentiation and other hormones augment its signaling pathway to make satisfactory adjustments in this process. After the cut is given, *wound induced dedifferentiation 1* (WIND1) stimulates cytokines which triggers the formation of callus at the graft junction. Simultaneously, auxin gets transported basipetally and due to the lack of vascular connectivity, its flow across the union is hampered, resulting in its accumulation above the graft interface and low concentration at the bottom junction. Auxin accumulation, along with ethylene signaling, triggers the expression of *Arabidopsis NAC domain containing protein* 71 (ANAC071) above the graft union, and simultaneously inhibits the expression of RAP2.6L as well as Jasmonic acid biosynthesis. Beneath the graft, the depleted auxin levels trigger the biosynthesis of Jasmonic acid and expression of RAP2.6L. The expression of ANAC071 and RAP2.6L prompts cell division around the graft junction. Auxin, in collaboration with gibberellins and cytokinins, stimulates differentiation of cells, resulting in formation of vascular connection and re-joining between both junctions, thereby restoring auxin symmetry. Gibberellins, in collaboration with auxin, stimulate emergence of tissues by means of cell expansion (Figure 7; Nanda and Melnyk, 2018).

## Effect of Rootstock-Scion Interaction on Growth, Quality and Stress Tolerance in Plants

Many reports recognized the interactions between several parameters related to physiology of the grafted trees and their fruit quality (Naor, 1998). These relationships are important since they provide a source for choosing the most compatible graft combination for specific environments and good fruit quality. Choice of a suitable combination is fundamental for increased production of trees, for the reason that the interaction between the stock and scion effects translocation of water and minerals, gas exchange in leaves, size of the plant, time and duration of flowering, time and percentage of fruit set and quality of fruits (Gonçalves et al., 2003). Therefore scion-stock combination is a key factor to be well thought-out in orchards before taking up the planting procedure. In grafted trees, the rootstock mainly controls the plant size. Performance of the fruit trees is fundamentally related to forming an optimum balance amongst growths and fruiting. Excessive vegetative growth always reduces the yield and deteriorates fruit quality (Mitre et al., 2012). Thus, for successful fruit production it is equally important to maintain an appropriate balance between the vegetative and reproductive processes (Sharma et al., 2009). Grafting scions on a less vigorous rootstock is the first and foremost step to achieve this equilibrium. In case of apple dwarfing rootstocks, it has been found that the sugar concentration is considerably low in these rootstocks and cellular action in these rootstocks is significantly reduced regardless of having huge starch reserves. Additionally, in dwarfing rootstocks, the down-regulation of *MdAUX1* and *MdLAX2* (auxin influx transporters) together with increase in flavonoids concentration lead to the reduced auxin movement which correlates to the dwarfing stature of these rootstocks (Foster et al., 2017). Lately, in apple *WRKY* transcription factor family has been found to be responsible for dwarfing phenotype in M26 rootstock. *MdWRKY9* on account of its differential expression in dwarfing and non-dwarfing rootstocks is considered as a candidate gene for controlling the dwarfing phenotype (Zheng et al., 2018). In addition to growth, many agronomically desirable qualities are also influenced by the pattern of stock-scion interaction. Pal et al. (2017) established that rootstock has a pronounced effect on growth as well as fructification in cherry. Lesser number of fruiting branches were formed in trees grafted on Mahaleb than those grafted on “Gisela 5.” A comparative evaluation of the two rootstocks indicated that fruit yield was almost double where “Gisela 5” was used as a rootstock compared to Mahaleb, in the first 3 years of fruiting. Rootstocks differ in their ability to utilize soil resources and transport these resources through the union to the scion. Owing to their different root architectures, rootstocks of grapevine and citrus differently take up phosphate and remobilize phosphorus reserves (Zambrosi et al., 2012; Gautier et al., 2018). Greater root length increases stomatal conductance in grafted grapevine under the conditions of water deficit (Peccoux et al., 2018). The influence of rootstocks on mineral concentration in scion leaf and fruits, plant growth, yield potential and quality traits of two apple cultivars was studied by Amiri et al. (2014). They found that rootstocks exert their influence on scion yield, quality and vigor by influencing the amount of minerals reaching the scion. M.9 rootstocks were more effective in nitrogen (N), manganese (Mn), and iron (Fe) uptake. The rootstock MM.106 was found to have the highest uptake potential for phosphorus (P) uptake, while M.9 had the lowest uptake potential for potassium (K) and calcium (Ca). One of the chief historic benefits of rootstocks is their ability to control diseases. These diseases can kill the plants at an early stage before they become productive or cause substantial impairment and yield reductions. The levels of disease resistance in a given cultivar can vary according to the rootstock onto which it is grafted (Cline et al., 2001). Jensen et al. (2012) measured the influence of rootstock on fire blight resistance of scion and observed that rootstocks predominantly influenced gala scion’s susceptibility to fire blight. Rootstocks were found to regulate the expression of different transcription factors. This differential gene expression pattern could be associated with variation in susceptibility. Besides increasing scion’s ability to deal with biotic stresses, rootstocks can also improve tolerance to a number of abiotic stresses. Amongst these, drought and high salinity have a massive control on crop production; certainly, they are one of the major aspects which restricts the plant productivity and effect yield severely (Cramer et al., 2007; Tsago et al., 2014). In grapevine the rootstocks “110R,” “1103P,” and “99R” have been found to enhance efficiency of using the water during critical stages of growth and as a result are effective in battling the drought stress (Nimbolkar et al., 2016). Grafting is the commercial method of propagation in citrus where the rootstock influences numerous horticultural traits, including tolerance to drought. A transcriptome analysis conducted in Sweet orange where Rangpur lime was used as the rootstock recommended that the rootstock was responsible for inducing the drought tolerance in scion cultivar by up regulating the transcription of genes associated to the cell wall, biotic and abiotic stress resistance, antioxidant systems and soluble carbohydrate, TFs, PKs, and *ABA* signaling pathway, and at the same time by down regulating the activity genes involved in the light reaction, metabolic processes and biosynthesis of ethylene (Gonçalves et al., 2019). In case of temperate fruits genetically diverse genotypes are available which serve as potential rootstocks against abiotic stress (Cimen and Yesiloglu, 2016). The interaction between the scion–rootstock has been reported to influence the quality and functioning in *Prunus avium*. The rootstock has been found to influence the movement of water and the process of photosynthesis in sweet cherry trees whereas the scion chiefly exerts its influence on other physical and chemical quality traits in cherry (Gonçalves et al., 2006). Santarosa et al. (2016) showed that the physiological interaction between rootstock-scion modified the vascular system in grapevines by altering the xylem vessel. Rootstock-scion combination that was highly vigorous had vessels with larger diameters, xylem areas, and proportion of vessels with diameters, greater than 50 μm. The effect of scion variety on rootstock growth and development via signals stimulated in shoot has been studied. These signals regulating root elongation and development in model plants include metabolites, hormones, peptides, HY5, microRNA 156, and microRNA 399. More study needs to be done to understand how scions modify phenotype of the rootstocks (Gautier et al., 2019). Tietel et al. (2020) studied the reciprocal effect between rootstock and scion, further they proposed that the interactions between the two could possibly be organ, time or distance dependent. Rootstock affected fruit yield and biochemical parameters of the fruit in relations with the scions. Overall distribution of 6 out of 53 and 14 out of 55 basic metabolites in the sap of scion and rootstock, was controlled by the rootstock, whereas 42 and 33 were affected by the rootstock-scion interaction, correspondingly.

## Molecular Responses at the Graft Interface

Presently, besides its application in horticulture, grafting has become more prominent as an important domain for research, chiefly regarding signaling mechanisms associated with rootstock and scion interaction. Recent studies have laid emphasis on detecting the transport of molecules particularly proteins and RNAs across long-distances. Proteins and RNAs transport across long-distances via phloem and their possible role in generating signals between the organs has turned to be a major field of research lately (Harada, 2010; Kehr and Buhtz, 2013).

### Movement of Genetic Components

Stegemann and Bock (2009) validated the movement of genetic material through the graft union and found that plastid genes travel small distances across the graft union. However, Stegemann et al. (2012) later found that the intact plastid genome moves across the graft union at the molecular level. Many genes on account of their role in hormones signaling are responsible for successful graft union formation. In *Arabidopsis*, it was found that the genes involved in wound healing and cleaning up of cellular debris were over expressed during the development of graft union (Yin et al., 2012). In grape vine, formation of the graft union triggered the differential pattern of expression of genes participating in secondary metabolism, modification of cell wall, and cell signaling (Cookson et al., 2013). The movement of the nuclear genome between the stock and scion led to the development of fertile alloploid plants (Fuentes et al., 2014). Phenotypic changes induced by grafting have led to the discovery of endogenous factors responsible for these changes. Initially the study was confined to anatomical aspects, nutrient and hormonal movement but now the latest technologies have shown that some mobile RNA molecules are transported via phloem tissue in the form of genetic material to complete the entire growth and development process. miRNAs, are a group of non-coding RNAs with roughly 22 nucleotides that control gene expression. These miRNAs, bring about either degradation of or inhibit translation of their target mRNAs (Bartel, 2004). Li et al. (2014) identified miRNAs in heterografts of vegetables, and observed that miRNAs played a critical role in controlling physiological processes in heterografts. Mo et al. (2018) identified miRNAs linked with graft union formation in Pecan and noticed the involvement of miRS26 in formation of callus and miR164, miR156, miRS10, miR160, and miR166 were found to be associated with differentiation of vascular bundles. These results suggest the role of miRNA in the successful graft union formation of pecan. Zhang et al. (2012) evaluated the movement of gibberellic acid insensitive (*GAI*) through a pear graft union. The results show that only 4 to 10 days after micro-grafting, Pyrus-*GAI* could be transported endogenously and not just this but it could also be transported to a scion of 10–50-cm height in a 2-year-old tree. The results serve as a basis for improving rootstocks and controlling scion properties in trees by the application of portable mRNA to fruit tree grafting. Agronomically significant traits such as compatibility, short juvenile period, dwarf phenotypes and antivirus can be transferred to the scion by making use of the transmissible mRNA in a transgenic rootstock (Rivera-Vega et al., 2011). Transport of *GAI-*mRNA in both directions between stock and scion has been reported in apple (*Malus* × *domestica* cv. Fuji and *Malus xiaojinensis*). Transport of *GAI* mRNA across the union took place 5 days after grafting, which points out the movement of *GAI* mRNA in both upward and downward direction (Xu et al., 2010). This study can serve as the basis for using RNA transport and its influence on properties of fruit trees. The study of complex rootstock-scion interactions in fruit trees is necessary for firming up resistance, higher yield and improving quality of fruits (Li et al., 2012). miRNAs have been known to influence several important stages of development like flowering time, response to various hormonal signaling, morphology of plant organs such as stem, leaves and roots (Pant et al., 2008; Xing et al., 2016). Moreover, miRNAs are also found to influence the interaction between the stock and scion. Earlier studies have revealed that miR398, miR395, and miR399 in the phloem are greatly linked with stress, and that the latter two are capable of moving from scions to rootstocks (Buhtz et al., 2008). Maturity in grafted avocado was found to be controlled by scion whereas rootstock encourages the successful union formation along with its influence on miRNA and mRNA profusion in the scion. The large amount of miR172, miR156 and the miR156 target gene SPL4, was directly associated with the scion and rootstock maturity in avocado (Ahsan et al., 2019). By comparing the expression of miRNAs in leaves and roots of homografts of cucumber seedlings, it was found that the expression of most miRNAs in the leaves and roots of heterografted seedlings altered vigorously: prompted under regular conditions and down regulated after some time of drought stress, and then again up regulated after 24 h of drought stress. These outcomes are valuable for the purposeful analysis of miRNAs in the facilitation of grafting induced drought tolerance. An et al. (2018) conducted a Gene Ontology study which showed that miRNAs which are expressed differentially throughout grafting regulated genes involved in a number of processes, including biosynthesis of cellular compounds and metabolism. Various studies revealed that miRNA172 can travel from source to sink in Nicotiana (Kasai et al., 2010) and that miRNA156 functions as a signal which is mobile through the phloem to affect important traits in potato (Bhogale et al., 2014). Lower expression levels of microRNAs (Vvi-miRNA159 and Vvi-miRNA166) were detected in more compatible combination compared to the less compatible. These microRNAs target the key TFs which promote plant growth and development (Assunção et al., 2019). It could therefore be said that it is the changes in RNA abundance brought about by grafting across the union that leads to differences in gene expression pattern resulting in changed phenotype. In cherry Zhao et al. (2020) detected more than 2 million sRNAs in each scion, among them 21-nt sRNA were the most abundant followed by 24-nt sRNA. On the other hand, 3000 sRNAs were transported from the scion into the rootstock. Out of these the most abundant ones were 24-nt sRNA followed by 21-nt sRNA. The study of sRNAs transported across the graft union provides a better understanding about their role in rootstock-scion interactions. Thus, the technique of grafting involves transport of genetic material across the union which facilitates further study of grafting mechanisms, rootstock scion interaction and the potential role of grafting in evolving new plant species (Wang et al., 2017).

### Movement of Proteins

**FIGURE 8:**
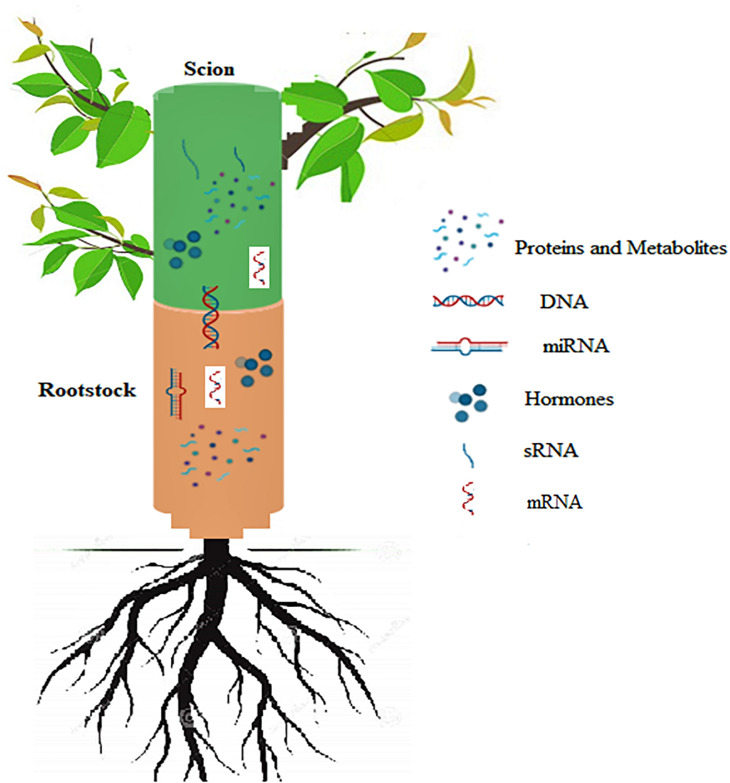
Schematic representation of signals exchanged between the rootstock and scion in grafted plants. DNA transfer across the graft union; Movement of sRNA across the graft union; Long distance transfer of miRNA and mRNA molecules; Hormonal signaling across the union; Movement of proteins and other metabolites through the graft union.

The phloem sap is known to contain mobile proteins which move across the graft union between the rootstock and scion. Long distance transport of proteins in plants across the graft union influences their growth and development by regulating some important processes, for example adventitious root formation in *Arabidopsis* is induced by the transport of *Arabidopsis* translationally controlled tumor protein 2 (AtTCTP2) protein across the graft union (Toscano-Morales et al., 2016). Besides their role in regulating plant growth and development, long distance transfer of proteins also plays an important role in combating different types of stresses. Proteomic analysis in cucumber scions grafted on *Momordica* rootstocks in response to heat stress revealed accumulation of 77 different proteins associated with important processes like photosynthesis, energy metabolism and synthesis of nucleic acids which eliminated the inhibitory effect of heat stress on scion growth (Xu et al., 2018). Buhtz et al. (2010) have shown that xylem vessels which generally transport water and solutes of low molecular weight contain proteins, even though at lesser concentrations compared to phloem. It has been reported that during transport from the site of production to sink tissues within the plants some proteins are able to bind with mRNAs as chaperones. These proteins can aid the transport and protect mRNAs from getting degraded. Duan et al. (2015) reported that polypyrimidine tract binding protein 3 (PbPTB3), which falls in PTB family of proteins and binds to a number of mRNAs in pear variety, Du Li (*Pyrus betulaefolia*) gets transported to long distances in the phloem and this process of transport is rather complex in nature controlled by the form of a tree, environmental conditions and nutrient concentration of the tree (Figure 8). There are other proteins which can move to long distances and regulate important cellular functions. For example, cyclophilin protein (SICyp1), a phloem-mobile protein was found to move from the scion to the stock through phloem. This transport accompanied by augmented auxin levels, eventually helped in promoting the root growth (Spiegelman et al., 2015). Paultre et al. (2016) reported protein trafficking in *Arabidopsis thaliana* micrografts, which displayed fluorescent protein-tagged signal peptides originally expressed in scion only, in the roots of rootstocks. This indicates extensive movement of proteins from the scion cells to the root cells. The study distinctly supports the existence of long-distance mRNAs and proteins transport, which can modify the physiological and morphological development of plants. The three TFs, VviLBD4, VviHB6, and VviERF3 involved in cambium activity, growth, and differentiation were found to be expressed differently between the two heterograft. A Transcriptomics study revealed that after 80 days of grafting expression of TF VviLBD4 is relatively high in compatible combination, together with VviHB6 and VviERF3. Since these TFs play a crucial role in maintaining the activity of cambial cells, growth, and tissue differentiation suggesting that in compatible combinations at 80 days after grafting, the transcription factors regulating cambial activity, cell differentiation and proliferation of callus are expressed at a higher rate. Furthermore, the results indicate that in the more compatible combination the expression of the above mentioned three TFs is substantially less reduced from 21 days after grafting to 80 days after grafting than in the less compatible ones (Assunção et al., 2019). In the same context, VviLBD4, VviERF3, and VviHB6 could serve as markers for the estimation of compatibility at the 80 DAG. Compatibility between the rootstock and scion is largely governed by the formation of callus cells at the union, which in turn is under the control of specific proteins. Differential expression of proteins at the graft union influences major biological functions including flavonoid synthesis. The up-regulation of proteins involved in flavonoid biosynthesis play a major role in callus formation during the healing of graft union (Xu et al., 2017) Another study reported enhancement of callus formation by the upregulation of the plant plasma membrane intrinsic protein (PIP1B), an aquaporin which increases water content of cells and promotes cell elongation resulting in successful union formation (Zheng et al., 2010). Studying mobility of proteins during grafting has shown that proteins are able to move from the companion cells of the shoot into the root cells and thereby regulating important physiological processes of plants (Paultre et al., 2016). This evidence will help us to improve our understanding of problems associated with grafting, successful graft union and perpetuation of horticultural crops, where the scion and stock material significantly affect the successful propagation and operative costs.

## Epigenetics in Grafting

The cells surrounding the graft intersection have to regulate under different metabolic, hormonal and redox conditions. These peculiar conditions might trigger epigenetic modifications and regulations which may play a role in graft healing. Some of the studies have shown that the epigenetic regulators are governed by redox status of cells (Shen et al., 2016; Berger et al., 2018) and are also associated with signals produced by hormones (Yamamuro et al., 2016). Plants face several kinds of stresses in the field conditions and these stresses have been also found to induce epigenetic modifications. Which contribute to plants adaptability, memory and productive response towards these stresses (Annacondia et al., 2018). There might be key epigenetic processes like DNA methylation and histone posttranslational modifications (PTM’S) governing some main events in successful graft, root stock interaction and healing (Probst and Mittelsten Scheid, 2015; Bilichak and Kovalchuk, 2016; Annacondia et al., 2018), *Brassica rapa* leaves were shown to undergo DNA methylation changes when subjected to the caterpillar *Pieris brassicae* (Kellenberger et al., 2016). Another example of the epigenetic modifications has been found to be induced in sweet potato caused by leaf crushing, which was found to induce small interfering RNAs (siRNAs), that were responsible to cause *LbMYB1* gene methylation RNA directed DNA methylation (RdDM) (Lin et al., 2013; Berger et al., 2018). Jasmonic acid (*JA*) linked with histone deacetylation and acetylation is a stress hormone produced at the graft interface. Histone deacetylases (HDACs) are induced by *JA*. Which suggest that PTMs and transcriptome reprogramming have a role in wound healing (Yamamuro et al., 2016; Zhang et al., 2016, 2017; Berger et al., 2018).

The discovery of non-autonomous RNA has made a significant contribution in understanding epigenetic modifications (Molnar et al., 2010; Bai et al., 2011; Tamiru et al., 2018; Gaut et al., 2019). In *Arabidopsis*, SRNAs were found to move from shoot to root and vice versa and were found to be linked with DNA methylation as they are the key components of the RdDM pathway (Law and Jacobsen, 2010). In genome wide methylation data large numbers (thousands) of methylated DNA bases were found within roots of methylation mutants (Lewsey et al., 2016; Gaut et al., 2019) signifying that sRNAs not only traverse graft junctions but also affect methylation in destination tissues. Epigenetic modifications and regulations have been suggested to play a role in callogenesis, a process which involves modifications in adult somatic cells from less differentiated states recuperating their capability for proliferation. Different models have been constantly deciphered to understand mechanisms of callogenesis and all suggest that it is also dependent on epigenetic regulations (Ikeuchi et al., 2013; de la Paz Sanchez et al., 2015; Birnbaum and Roudier, 2017; Lee and Seo, 2018).

## Conclusion

Despite the fact that grafting technology has improved enormously, the long-standing survival of grafted plants is still to a certain extent unpredictable due to the incompatibility problems. However, a prior selection method can serve as an efficient means to foresee the future of a compatible combination way before any external symptoms appear. The effect of rootstock-scion interactions pertaining to growth, attainment of reproductive potential, fruit set, yield efficiency and quality characteristics of fruit crops is complex and poorly understood. A healthier understanding of rootstock scion interactions would aid in more effective selection and use of rootstocks in future. The latest technology of silencing transmissible RNA and its potential to regulate growth and stress responses has provided new opportunities to understand stock-scion relationships. On the basis of the results of different studies discussed in this review, it is now possible to make use of mobile mRNAs in grafting systems of fruit trees. Undeniably, there is great scope for the development of transgenic rootstocks, in fruit trees that carry transportable mRNAs which regulate key horticultural traits, such as disease and stress resistance properties or dwarfing growth habit. This kind of approach would not only improve the characteristics of scion using transmissible mRNA from a transgenic rootstock, but also might shun some of the disagreements regarding transgenic fruit production. The epigenetic modifications occurring at the graft site is one of the most important yet unexplored fields. Understanding methylation patterns, epigenetic markers and maintenance of these changes in different perennial crops is a very important field to explore in future research.

## Author Contributions

AR and SM: conceptualization. AR and TB: software. GIH, KMB, MNA, PA, and AJ: validation. AR and SM: writing—original draft preparation. AR, SM, KMB, GIH, BAP, AAA, and PA: writing—review and editing. AR, SM, GIH, HAE-S, BAP, and PA: supervision. All authors contributed to the article and approved the submitted version.

## Conflict of Interest

The authors declare that the research was conducted in the absence of any commercial or financial relationships that could be construed as a potential conflict of interest.
